# Double-Blind, Randomized, Phase 2a Study to Evaluate the Safety and Immunogenicity of OVX836, a Nucleoprotein-Based Universal Influenza Vaccine, Co-Administered with Fluarix Tetra^®^ or Afluria Quad^®^

**DOI:** 10.3390/vaccines14100860

**Published:** 2026-09-28

**Authors:** Nicola Groth, Jacques Bruhwyler, Emilie Piat, Jessika Tourneur, Philippe Moris, Michael Penlington, Florence Nicolas, Alexandre Le Vert

**Affiliations:** 1 Clinical Department, Osivax, 7 Rue des Chasseurs Ardennais, Angleur, 4031 Liège, Belgium; ngroth@osivax.com (N.G.); philippe.moris@hotmail.com (P.M.); mcpenlington@gmail.com (M.P.); 2 Osivax Headquarter, 70 Rue Saint-Jean-de-Dieu, 69007 Lyon, France; epiat@osivax.com (E.P.); jessikatourneur@gmail.com (J.T.); fnicolas@osivax.com (F.N.); alevert@osivax.com (A.L.V.)

**Keywords:** nucleoprotein, OVX836, influenza vaccine, universal vaccine, HA immune response, anti-nucleoprotein IgG humoral response, NP-specific T-cell immunity

## Abstract

**Background/Objectives:** For decades, influenza vaccine development has focused on eliciting antibody responses. Increasing evidence suggests that cell-mediated immune (CMI) responses can complement the humoral response by providing broader protection. This study evaluated the safety and immunogenicity of an experimental nucleoprotein (NP)-based vaccine, OVX836, when co-administered with a licensed hemagglutinin (HA)-based inactivated split influenza vaccine (IIVs; either Fluarix Tetra^®^ [Fluarix] or Afluria Quad^®^ [Afluria]). **Methods:** This randomized, double-blind, phase 2a study (ClinicalTrials.gov: NCT05734040) included six groups, each of approximately 80 healthy adults (18–60 years old). Participants received either a) OVX836 (480 μg) concomitantly administered with Fluarix, Afluria, or placebo, b) Fluarix or Afluria concomitantly administered with placebo, or c) double placebo (double-dummy approach). Treatments were administered concomitantly by intramuscular injection into the deltoid muscles of opposite arms. Endpoints included reactogenicity, safety, and immunogenicity, measured by hemagglutination inhibition assay (HAI), anti-NP immunoglobulin G (IgG), and NP-specific CMI. **Results:** Concomitant administration of OVX836 with Fluarix or Afluria was safe and well tolerated. HAI responses to the IIV strains were similar when administered with OVX836 or placebo. Groups receiving OVX836 demonstrated markedly enhanced humoral anti-NP IgG and NP-specific CMI responses, which were negligible in the non-OVX836 groups. No evidence of clinically significant immunological interference between OVX836 and IIVs was observed for any endpoint. **Conclusions:** Concomitant administration of OVX836 did not adversely affect IIV safety or HAI responses, while inducing robust anti-NP IgG and NP-specific CMI responses. These findings support further clinical development of OVX836 in combination with licensed IIVs.

## 1. Introduction

Influenza alone contributes to approximately 290,000 to 650,000 fatalities annually [1], depending on the circulating virus strains each season. As with other respiratory infections, influenza disease is particularly severe in vulnerable populations, including young children, the elderly, and individuals of any age with weakened immune systems.

Vaccine efficacy substantially varies from season to season [2], ranging from 10% to 60%, depending on antigenic diversity of circulating strains [3]. When well matched to circulating strains, influenza vaccine effectiveness typically ranges from 40% to 60% [4]. These levels fall short of the ~70% efficacy threshold, assuming at least 75% population coverage, required to prevent an epidemic [5].

Influenza vaccine development has traditionally targeted protective hemagglutinin (HA) antibody responses. However, mounting evidence that T-cell immunity also contributes to protection presents an important opportunity [6]. Vaccines designed to induce both broad T-cell effector functions and robust antibody responses may therefore offer enhanced protection [7,8], particularly to vulnerable populations, or when the vaccine antigens and circulating strains are poorly matched.

The OVX836 influenza vaccine candidate, developed by Osivax (Lyon, France), employs a recombinant protein approach that leverages the full-length nucleoprotein (NP) from the A/WSN/1933 (H1N1) strain. NP is a structural protein critical for viral RNA transcription, replication, and genome packaging, and is highly conserved among influenza A strains, with over 90% sequence homology across subtypes [8,9]. Targeting the well-conserved internal NP may facilitate development of influenza vaccines with broader protection through stimulating the cellular immune response. Furthermore, unlike the highly variable surface proteins HA and neuraminidase (NA), which undergo frequent antigenic drift and shift, NP evolutionary stability makes it an attractive candidate to consistently stimulate T-cell–based immunity. For example, CD8+ T cells exert direct cross-protective cytotoxic effects on infected cells in the lung, thereby restricting viral replication, whereas CD4+ T cells perform diverse effector functions, including cytokine secretion to support antibody production [10,11,12,13]. Studies in both humans and animal models have demonstrated that strong NP-specific T-cell responses correlate with reduced viral load and milder influenza disease [14]. By incorporating conserved internal antigens such as NP, next-generation influenza vaccines could potentially offer more durable, wider protection against both seasonal and pandemic strains.

The proprietary self-assembling domain OVX313 (oligoDOM^®^) of the vaccine candidate builds on prior work demonstrating self-assembly properties [15]. The immunogenicity of NP was subsequently confirmed when fused to OVX313 [16]. In this fused form, OVX313 drives the spontaneous assembly of nanoparticles, each displaying seven full-length NP molecules in a multimeric, highly immunogenic configuration. This architecture enhances immune recognition, particularly against conserved internal influenza antigens.

The safety, humoral and cellular immune responses of OVX836 have been evaluated in several Phase 2a trials within the 18- to 65-year age range [17,18]. Across all studies, OVX836 (180 µg, 300 µg and 480 µg) induced robust humoral (anti-NP IgG) and cellular immune responses, as evidenced by increased IFNγ spot-forming cells (SFC) per million PBMCs and CD4+ T cells expressing IFNγ. These NP-specific responses were highly cross-reactive against multiple influenza A strains, including seasonal and highly pathogenic avian subtypes [18]. CD8+ T-cell responses were observed at the 300 µg and 480 µg doses in one trial on participants aged 18 to 55 years [17]. Across all studies, OVX836 exhibited a favorable safety profile up to 480 µg, the maximum dose tested. There was no dose-dependent increase in reactogenicity and no evidence of reaching a maximum tolerated dose.

In a subsequent study (OVX836-004), the immune response to OVX836 concomitantly administered with the licensed tetravalent inactivated influenza vaccine (IIV) Fluarix^®^ Tetra (hereafter Fluarix) was comparable to when Fluarix was concurrently administered with placebo [19]. These concomitant injections were administered in the same deltoid muscle to maximize the likelihood of detecting lymph node–mediated interference. In contrast to groups vaccinated only with Fluarix and placebo, Fluarix concomitantly administered with OVX836 induced significant T-cell responses.

The current Phase 2a (OVX836-006) study was designed to broaden the applicability of the findings of the parent (OVX836-004) study [19]. Safety, HA-specific and anti-NP IgG humoral response, as well as NP-specific T-cell response were evaluated in a larger non-elderly adult population (up to 60 years of age) following concomitant administration of OVX836 with one of two licensed IIVs. In contrast to the parent study, vaccines were injected into opposing deltoid muscles. The study also aimed to confirm that OVX836 did not significantly impair the humoral response to IIVs, and that IIVs did not interfere with the NP-specific T-cell or anti-NP IgG response induced by OVX836, based on coadministration with either of the two licensed IIVs.

## 2. Materials and Methods

This randomized, double-blind (double-dummy), parallel-group Phase 2a study (OVX836-006; ClinicalTrials.gov NCT05734040; Registration date: 17 February 2023) was conducted from 10 May 2023 to 15 January 2024 in compliance with Good Clinical Practice (GCP) at nine investigational sites in Australia. The study received approval from the Australian Human Research Ethics Committees and acknowledgment through the Clinical Trial Notification process by the Australian Therapeutic Goods Administration. All participants provided written informed consent.

The sample size estimation was based on CPMP/BWP/214/96 (1997) requirements and previous methodology as described by Groth et al. [19]. The study planned to enroll a total of 600 healthy male and female participants aged 18 to 60 years. Pregnant women (positive urine test on Day 1) and breastfeeding individuals were excluded. All participants had received at least one dose of a licensed SARS-CoV-2 (COVID-19) vaccine according to national recommendations. Detailed eligibility criteria are available in Supplementary File S1. Participants were randomized at a 1:1:1:1:1:1 ratio into six treatment groups.

Participants in three of the six groups were administered OVX836 (480 µg) concomitantly with either Fluarix^®^ Tetra (Fluarix), Afluria Quad^®^ (Afluria), or a placebo (NaCl 0.9%). In two other groups, Fluarix or Afluria was co-administered with placebo, while the final group received two placebo (double dummy) injections. All injections were administered intramuscularly (IM) into the deltoid muscles of opposite arms.

Fluarix and Afluria are purified split IIVs licensed and commercially available in Australia, which, in the 2023 influenza season, comprised 15 µg each of A/Sydney/5/2021 (H1N1)pdm09-like virus, A/Darwin/9/2021 (H3N2)-like virus, B/Austria/1359417/2021 (B/Victoria lineage)-like virus and B/Phuket/3073/2013 (B/Yamagata lineage)-like virus). The placebo consisted of NaCl 0.9%.

The study was double-blind for treatment allocation. Due to the differing appearance of the vaccines, an unblinded nurse prepared and administered the study treatments.

A subset of participants was selected for peripheral blood mononuclear cell (PBMC) sampling to assess the cell-mediated immune (CMI) response. This subset consisted of approximately 50% of those receiving OVX836 (up to 150 subjects) and 10% of those in the placebo or Fluarix/Afluria + placebo groups (up to 30 subjects), based on the randomization scheme. This subset was exclusively recruited from urban centers, as rural centers lacked proximity to laboratories with adequate sample processing capacity.

After the injection on Day 1, participants were kept under observation in the investigational center for 30 min, followed by a 7-day period when solicited local (injection site pain, redness and swelling) and systemic (fatigue, headache, arthralgia, malaise, myalgia and fever) symptoms were collected. While all local symptoms were considered related to the study treatment, solicited systemic symptoms were assessed for causality by the Investigator. All unsolicited adverse events (AEs) were reported for a 28-day period post-injection and serious adverse events (SAEs) were reported during the entire study period (until Day 180). The severity of AEs was assessed in accordance with the Food and Drug Administration’s guidance (2005) on the Toxicity Grading Scale for Healthy Adult and Adolescent Volunteers Enrolled in Preventive Vaccine Clinical Trials.

The humoral immune response was assessed in all participants on Day 1 and Day 29, and additionally in the CMI subset (maximum 150 and 30 participants in the OVX836 and non-OVX836 groups, respectively) on Day 8, when anti-NP IgG titers were also assessed.

Participants who were selected for assessment of the HA humoral response only (i.e., not in the CMI subset) had a total of 20 mL of whole blood drawn: 10 mL each on Day 1 and Day 29. The subset of participants selected to assess the CMI response in addition to humoral response donated approximately 130 mL of whole blood: 60 mL on Day 1, 60 mL on Day 8, and 10 mL on Day 29. About 50 mL of whole blood was used to isolate PBMCs for evaluation of NP-specific T-cell-mediated immunity (Day 1 and Day 8), and 10 mL of whole blood was used to collect serum for humoral immunity assessment (Day 1, Day 8, and Day 29). The immunoassays are described in Supplementary File S2.

Participants with influenza-like illness (ILI) tracked their symptoms using a standardized questionnaire (Flu-PRO^©^) during the entire study. Participants performed nasal swabs at home and, within three days of symptom onset, presented at a clinic where an additional nasopharyngeal swab was taken. Both nasal swabs were analyzed for influenza A and B viruses and SARS-CoV-2 by reverse transcriptase-polymerase chain reaction (RT-PCR).

The primary safety objective evaluated safety and reactogenicity of OVX836 when concurrently administered with IIVs or with placebo into opposite arms. The primary immunogenicity objective evaluated the HA immune response against each of the four influenza strains elicited by the two IIVs, when each was concomitantly administered with OVX836 or placebo.

The primary immunogenicity criteria were assessed according to the *Committee for Medicinal Products for Human Use* thresholds for seasonal influenza vaccine approval (Note for Guidance on Harmonization of Requirements for Influenza Vaccines, CPMP/BWP/214/96, 1997). At least one of the following assessments should be met for each strain: (i) a negative pre-vaccination HAI titer and post-vaccination HAI titer ≥1:40, or a fourfold increase in HAI titer between pre- and post-vaccination timepoints (seroconversion rate); (ii) ≥70% of subjects achieving a HAI titer ≥ 1:40 at Day 29 (seroprotection rate); and/or (iii) Day 29/Day 1 HAI titer geometric mean ratios (GMRs) > 2.5. According to the Food and Drug Administration’s (FDA) Guidance for Industry, Clinical Data Needed to Support the Licensure of Seasonal IIVs (2007), the lower limit (LL) of the 95% confidence interval (95% CI) had to be above 40% for the seroconversion rate and 70% for the seroprotection rate.

The secondary immunogenicity objectives were: (i) the HAI geometric mean titers (GMTs) at baseline and at Day 29 for each of the four IIV strains; (ii) the change in NP-specific T-cell frequencies in PBMCs, measured by IFNγ ELISPOT versus pre-injection baseline (Day 1), (iii) the GMTs of anti-NP IgG on Day 1, 8 and 29; (iv) the number and percentage of subjects with a four-fold increase in anti-NP IgG titer on Day 29 over baseline (Day 1). A further secondary objective evaluated absolute and relative vaccine efficacy.

The percentages of NP-specific CD4+ and CD8+ T cells expressing IFNγ, IL-2 and/or TNFα, at baseline (Day 1) and Day 8 were assessed by flow cytometry as an exploratory endpoint.

The safety analysis set included all participants, as treated, who were administered at least one of the two injections and had any post-vaccination safety data.

The modified intention-to-treat (mITT) analysis set included participants from all groups who were administered two injections, excluding all Site 01 subjects due to protocol violations. The Per-Protocol Day 29 (PP-D29) and Day 180 (PP-D180) analysis sets included all participants who received 2 injections, donated evaluable baseline and post-administration blood samples up to Day 29 or Day 180 (including the CMI subset), and without significant protocol deviations. 

The sample size justification and statistical methodologies are exhaustively described in Supplementary File S3.

## 3. Results

### 3.1. Participant Disposition

A total of 477 participants were enrolled and received a study vaccine and/or placebo. Between 76 and 83 participants were assigned to each of the six treatment groups (Figure 1). A total of 439 participants completed the study.

The mITT, PP-D29, and PP-D180 analysis sets comprised 419, 378, and 351 participants, respectively. Safety was analyzed in the whole population, while immunogenicity was analyzed in both the mITT and PP populations. Site 01 was excluded from both mITT and PP analyses based on concerns of systematic deviations from study procedures following an internal audit. The analyses are presented for the PP population only, as it represents the more conservative approach. In total, 202 participants were analyzed in the full PP-D29 set and 86 in the CMI subset of the three groups that concomitantly received OVX836. A total of 176 participants were analyzed in the full PP-D29 set and 16 in the CMI subset of the 3 groups that did not concomitantly receive OVX836 (i.e., received IIV and/or placebo).

### 3.2. Study Population Demographics and Baseline Characteristics

Overall, participants were 38.8 ± 12.9 years old (mean ± SD), 50.3% were female, and the majority were White-Caucasians (78.0%). Other races/ethnicities were principally Asian (16.1%) and Hispanic/Latino (3.8%). Although there was a borderline difference (*p* = 0.050; ANOVA) among the six groups of the safety analysis set for BMI (range, 26.5 to 28.4), this was not clinically significant and, generally, baseline characteristics were balanced across the six treatment groups of the safety analysis set (Table 1).

Demographic and other baseline characteristics were comparable among the safety, mITT, PP-D29, and PP-D180 groups, with no major differences in sex, age, race, smoking status, height, or weight.

### 3.3. Reactogenicity and Safety

There was a tendency for slightly higher percentages of participants experiencing pain in the three groups that concurrently received OVX836 than in the groups administered Fluarix or Afluria with the placebo, or placebo only. No recipient of OVX836 experienced a severe local reaction. One subject reported severe pain (Grade 3) in the Fluarix + placebo group. Redness and swelling were experienced in under 5% of each group and appeared balanced across treatment groups (Table 2).

Overall, local reactions were observed at 55.6% of OVX836 injection sites (N = 248 OVX836 doses), regardless of which concomitant vaccination was given in the opposite arm. This was comparable to the incidence of local reactions at any of the injection sites of Fluarix (59.1% [N = 159 Fluarix doses]) or Afluria (56.0% [N = 159 Afluria doses]), but higher than for placebo (32.0% [N = 388 placebo doses]). The duration of local reactions (2 to 3 days) was comparable among all groups.

The percentages of participants reporting systemic symptoms to OVX836, whether concurrently administered with Fluarix, Afluria, or placebo, were similar to those when Fluarix or Afluria was concurrently administered with placebo, and when placebo was administered alone (Table 2). In the safety analysis set, headache (24.4% to 53.9% of subjects) and fatigue (41.5% to 51.3% of subjects) were the only systemic symptoms reported by more than half of the participants in any group, with no consistent trend across groups, including when only placebo was administered. Each systemic symptom categorized as severe (grade 3) occurred in no more than one participant in any group and appeared evenly distributed among groups. Most systemic solicited symptoms were considered related to the study treatments and of short duration, generally lasting 1 or 2 days.

The percentages experiencing any (range, 20.5% to 31.2%) or possibly related (range, 3.6% to 8.4%) unsolicited adverse events in the groups receiving OVX836 and/or IIVs were similar to the placebo group (28.9% and 10.5%, respectively). The majority were mild or moderate in severity. Infections and infestations were the most affected system organ class (range: 6.1% to 14.3%).

A total of 16 SAEs were reported for 16 subjects, none of which was considered related to the vaccines and/or placebo.

### 3.4. Immunogenicity

#### 3.4.1. Primary Immunogenicity Endpoint (HAI Responses)

At Day 29, at least one of the three HAI Committee for Medicinal Products for Human Use (CHMP) criteria (seroconversion rate of at least 40%, 70% of subjects achieving a seroprotective titer ≥1:40 and/or GMR > 2.5) was achieved for A/Sydney/5/2021 (H1N1), A/Darwin/9/2021 (H3N2) and B/Austria/1359417/2021 (B/Victoria lineage) influenza strains in the groups receiving the IIVs, regardless of whether concomitantly administered with OVX836 or placebo (Table 3). For the B/Phuket/3073/2013-like strain (B/Yamagata lineage), only one of the three CHMP criteria was met when (i) Fluarix was administered with placebo or (ii) Afluria was administered with either OVX836 or placebo. Fluarix administered with OVX836 did not meet any of the three CHMP criteria for the B/Phuket/3073/2013-like strain.

In addition, as required by the corresponding FDA criteria, the lower limit of the 95% CI around midpoint seroprotection rates was above the threshold of 70% for A/Sydney/5/2021 (H1N1) when either of the IIVs was only concomitantly administered with placebo but not OVX836, and for A/Darwin/9/2021 (H3N2), regardless of whether administered concomitantly with placebo or OVX836. The lower limit of the 95% CI around midpoint seroconversion rates was above the lower 40% limit, except for the B/Austria/1359417/2021 (B/Victoria lineage) when Fluarix was concomitantly administered with OVX836. The FDA seroconversion criterion was not achieved for any group against the B/Phuket/3073/2013-like (B/Yamagata lineage) strain (Table 3).

#### 3.4.2. Secondary Immunogenicity Endpoints

##### HAI GMTs

Baseline (Day 1) HAI GMTs were balanced across the six treatment groups (*p* > 0.05; ANOVA). OVX836 administered with placebo, or placebo administered alone, did not induce any HAI antibodies. At Day 29, highly significant HAI responses were measured for all strains in all IIV groups, with or without OVX836 (Figure 2).

No statistical differences between GMTs against all four vaccine strains at Day 29 were found between recipients administered Fluarix and OVX836 or Fluarix and placebo (*p* > 0.05; ANOVA). Similarly, Day 29 GMTs did not significantly differ when Afluria was administered with either OVX836 or placebo (*p* > 0.05; ANOVA).

When Fluarix was administered with placebo, the increase in GMT at Day 29 over Day 1 (i.e., GMR) was statistically significantly higher for the A/Sydney (H1N1) strain (*p* = 0.007; ANOVA and Bonferroni’s tests) and the B/Austria strain (*p* = 0.047) than when it was administered with OVX836.

##### NP-Specific IFNγ SFC Measured by ELISPOT

The CMI subset comprised between 26 and 32 participants from each of three groups concomitantly administered OVX836 versus between 4 and 7 participants for the three groups that were not administered OVX836. No anti-NP CMI responses, as measured by IFNγ ELISPOT, were detected at Day 8 when the IIVs were concomitantly administered with placebo, or when only placebo was administered (Figure 3). When OVX836 was administered with Fluarix, it induced a significantly stronger anti-NP CMI response (as measured by IFNγ ELISPOT) than when Fluarix was administered with placebo (*p* < 0.01, ANOVA) or when placebo was administered alone (*p* < 0.05, ANOVA). In contrast, although the Day 8 anti-NP CMI response to OVX836 administered with Afluria was higher than when Afluria was administered with placebo or placebo was administered alone, this difference did not attain statistical significance (*p* > 0.05, ANOVA).

The mean number of NP-specific IFNγ spot-forming cells (SFCs) per million PBMCs between Day 1 and Day 8 increased by 125 ± 135 in the Fluarix + OVX836 group (3.30-fold increase), 111 ± 154 in the Afluria + OVX836 group (4.30-fold increase), and 132 ± 167 (4.40-fold increase) in the OVX836 + placebo group. No statistically significant difference was found between the three groups administered OVX836 (*p* > 0.05, ANOVA).

##### Humoral Anti-NP Immune Response

No statistically significant differences (*p* > 0.05; ANOVA) were found in baseline anti-NP IgG GMTs (range, 4031–4287) among the 6 treatment groups. As expected, the IIVs administered with placebo and the placebo alone did not induce any anti-NP-specific humoral immune response (Figure 4). OVX836 administered with Fluarix, Afluria, or placebo induced highly statistically significant increases in GMTs at Day 8 (*p* < 0.001; paired Student’s t tests), which further increased at Day 29 (*p* < 0.001) to 17,334 (GMR = 4.04), 19,444 (GMR = 4.56), and 22,081 (GMR = 5.48), respectively. The difference between these three groups was not statistically significant (*p* > 0.05; ANOVA) at Day 8. However, at Day 29, the anti-NP IgG GMT was significantly higher when OVX836 was concomitantly administered with the placebo (22,081) than with Fluarix (17,334) (*p* = 0.039; ANOVA and Bonferroni’s tests), but not between the other groups concomitantly administered OVX836.

##### Four-Fold Increases in Anti-NP IgG Titers over Day 1

On Day 29, 68.8%, 76.2% and 76.0% of the participants administered OVX836 concomitantly with Fluarix, Afluria or placebo (*p* > 0.05 for intergroup differences; chi-square test) achieved at least a 4-fold increase in the anti-NP IgG titers, which were significantly higher (*p* < 0.001; chi-square test) than the 14.0% and 19.3% of the participants administered Fluarix or Afluria concomitantly with placebo, and 0% when placebo was administered alone.

##### NP-Specific CD4+/CD8+ T Cell-Mediated Immune Response Measured by Flow Cytometry

When the IIVs were administered with placebo, or placebo was administered alone, no increase in NP-specific CD4+ cells expressing any of the three cytokines (IL2+, TNFα-, IFNγ) or cytokine combinations was detected at Day 8 compared with Day 1 (*p* > 0.05; Friedman’s test [Figure 5]). When OVX836 was administered with Fluarix, Afluria or placebo, statistically significant increases in the percentages of NP-specific CD4+ T cells between Day 1 and Day 8 were observed for cells expressing at least one cytokine (*p* < 0.01; Friedman’s test), at least IFNγ (*p* < 0.01), IFNγ+ only (*p* < 0.01), IL2+/TNFα-/IFNγ+ (*p* < 0.01) and polypositive (at least two of the three cytokines; *p* < 0.05). Day 8 increases from baseline in the percentages of NP-specific CD4+ T cells in groups administered OVX836 concomitantly with placebo, Fluarix, or Afluria were not statistically significant (*p* > 0.05; Kruskal–Wallis tests).

There was no statistically significant increase in the percentages of NP-specific CD8+ T cells in the six treatment groups of this study (*p* > 0.05; Friedman’s tests).

##### Influenza-like Illnesses

The total number of RT-PCR-confirmed influenza A cases occurring more than 14 days post-vaccination (N = 7) was too limited (global attack rate in all 6 groups = 7/477 = 1.47%) to be able to conclude any absolute or relative VE.

## 4. Discussion

The safety and tolerability profiles of OVX836 (480 µg), the two IIVs (Fluarix and Afluria), and OVX836 (480 µg) administered concomitantly with either IIV in opposite deltoid muscles were comparable. These profiles were consistent with previously published results for OVX836 administered alone [17,18] or concomitantly with Fluarix in the same deltoid muscle [19]. When either IIV was administered with OVX836, rates of injection-site pain were marginally higher compared with concomitant injection of IIV with placebo; however, these differences were neither clinically meaningful nor statistically significant, and no cases were severe.

Across all six treatment groups, including when placebo was administered alone, the incidence of systemic symptoms was generally higher than that reported in the IIV prescribing information (e.g., fatigue was reported by 47.0% of Fluarix + OVX836 recipients and 43.3% of the placebo-only recipients of the current study vs. 16% of Fluarix recipients in the prescribing information [20]). While this may reflect extrinsic considerations between studies (e.g., phase 2a vs. large late-stage phase 3), the percentages of participants reporting systemic symptoms were balanced across treatment groups of this study, with no indication of more frequent or more severe reactions when OVX836 was administered. Unsolicited AEs were primarily categorized as infections and infestations, or were known common side effects of vaccination, and were expected in this population. None of the 16 SAEs reported during the study were considered related to study treatments.

The CHMP criteria for seroprotection, seroconversion, and GMRs at Day 29 against the A strains were met in groups administered IIVs (with and without OVX836). While influenza A components of vaccines typically elicit stronger immune responses than B components [21] due to their higher intrinsic immunogenicity and the greater degree of immune priming from repeated exposures [22,23], the IIVs also induced a robust response against the B/Victoria lineage, as demonstrated by the attainment of all CHMP seroconversion and GMR criteria by all four IIV groups. The more stringent FDA criteria showed a similar pattern, however, and were met more frequently for the A strains than for B/Austria/1359417/2021.

Although HA responses were weakest against the B/Phuket/3073/2013-like strain, the CHMP requirement for GMR was met in three of the four IIV groups, with only Fluarix co-administered with OVX836 failing to achieve a GMR increase above 2.5. Therefore, three of the four IIV groups satisfied the primary objective that at least one CHMP requirement be met for each strain. The failure of the Fluarix + OVX836 group to meet this objective for the B/Phuket/3073/2013-like strain is more likely attributable to chance than to clinically meaningful interference by OVX836, given that: (i) GMT responses to this strain were generally lower than to other strains across all four groups; (ii) differences in GMRs between groups were small (range, 2.13–2.75); (iii) GMRs in Afluria recipients were similar regardless of whether concomitantly administered with OVX836 or placebo and (iv) the parent study (OVX836-004) found no difference in GMRs when Fluarix was administered with or without OVX836 [19]. Although the reason for the weaker HA response of IIVs against the B/Phuket/3073/2013-like strain compared with the prior study is unclear, annual variation in IIV responses to B/Yamagata lineage strains is well documented. Moreover, the absence of confirmed circulation has prompted discussion on phasing out B/Yamagata lineage strains in influenza vaccines, including B/Phuket/3073/2013, as these viruses have not circulated since 2020 [24].

Although OVX836 elicited higher fold increases in the number of NP-specific IFNγ SFCs per million PBMCs at Day 8 when administered with Fluarix, Afluria, or placebo compared with the corresponding treatments without OVX836, statistical significance was achieved only between the two Fluarix groups. It is important to note that the sample sizes for the groups without OVX836 were small (N = 4–7), which limited the power to detect differences. Moreover, the mean fold increase observed for Afluria + OVX836 compared with Afluria + placebo was greater than the corresponding difference for Fluarix, despite the lack of statistical significance.

OVX836 administered with Fluarix, Afluria or placebo significantly increased anti-NP IgG levels at Day 8 compared with Fluarix or Afluria administered with placebo. This is consistent with previous findings [19] when OVX836 concomitantly administered with Fluarix induced a significantly greater response than Fluarix administered with placebo. In the current and previous studies, further increases were observed by Day 29. Although anti-NP IgG lacks direct neutralizing activity, mechanisms such as antibody-dependent cellular cytotoxicity and antibody-dependent cellular phagocytosis mediated by natural killer cells [25], potentially through Fc-dependent binding to virus-infected cells, may contribute to protective efficacy. The relevance of these pathways to the clearance of influenza virus–infected cells, and therefore to protection, remains hypothetical and speculative. Nevertheless, the involvement of IgG against this internal antigen of the influenza virus in antibody-dependent cellular cytotoxicity, immune complex–mediated NK cell priming, or related mechanisms suggests a plausible OVX836-enhanced response that merits further investigation [17,18].

The NP-specific responses of CD4+ T cells expressing IFNγ, IL-2, TNFα, or any combination of these cytokines showed statistically significant increases at Day 8 when OVX836 was administered with Fluarix, Afluria, or placebo. Such NP-specific IFNγ-secreting T cells have been associated with cross-reactive cellular immunity against both influenza A and B strains, facilitating rapid viral clearance even when neutralizing antibody titers are suboptimal [26]. These findings indicate the potential of this vaccine candidate to enhance protection when concomitantly administered with vaccines known to have a protective antibody response against influenza. This is especially consistent with Groth et al. [19], considering that OVX836 does not appear to significantly interfere with that response.

In contrast to Leroux-Roels et al. [17], but consistent with the findings of Groth et al. [19], OVX836 did not appear to elicit CD8+ T-cell responses. Epidemiological and demographic variables in the populations may account for this apparent difference. The studies were conducted in different years, in opposite hemispheres, and at varying periods relative to the influenza season. Exposure history could prime or constrain CD8+ responses [27]. In addition, there was a sex imbalance towards females (71%) in the study of Leroux-Roels et al. [17], versus 59.4% in the study of Groth et al. [19] and 50.3% in the current study. Generally, females tend to mount stronger humoral and cellular immune responses to OVX836 than males [18], which may also have contributed to the observed outcomes.

Only limited differences in HAI, CMI, or NP responses were observed between the parent study, where OVX836 was co-administered with Fluarix into the deltoid muscle of the same arm [19], and the present study. This finding suggests that there is no significant immunological interference, whether vaccine drainage is through the same or different lymph nodes. These findings also confirm immunogenicity and safety when vaccinations are given in opposite arms.

Overall, the results of this Phase 2a study, in which the study treatments were administered into opposing deltoid muscles, which involved a larger population and included 55- to 60-year-old adults, are consistent with those of the smaller previous study [19]. Both studies provide converging evidence that co-administration of OVX836 with licensed IIVs did not, or only marginally, interfere with humoral immune responses to the IIVs, while enhancing a non-neutralizing antibody response against the NP. In addition, OVX836 significantly boosted the cellular immune response, with no evidence of diminished responses when co-administered with either of the two IIVs.

The main limitation of this study was the slow enrollment of participants. The original target was approximately 600 subjects; however, only 477 were enrolled. A total of 378 subjects were included in the Day 29 per-protocol population after exclusion of Site 01. This reduction limited the ability to meaningfully interpret analyses stratified by age group (18–44 and 45–60 years). One further limitation was that the CMI subset included only a small number of participants, the majority of whom were assigned to the OVX836 groups, and all were recruited from urban centers near the laboratories where the swabs were analyzed. This may have influenced both the observed occurrence of ILI and the assessment of CMI.

## 5. Conclusions

OVX836 480 µg was safe and well tolerated when administered intramuscularly, either alone (with placebo) or concomitantly with Fluarix^®^ Tetra or Afluria Quad^®^, in opposing deltoid muscles. Both licensed IIVs induced robust HAI antibody responses regardless of whether concomitantly administered with OVX836 or placebo. OVX836, in addition to generally preserving humoral immune responses to the licensed IIVs against the circulating strains, significantly enhanced NP-specific CMI responses. Such responses, to be interpreted cautiously given the limited size and imbalance of the corresponding study subgroups, may be particularly important in helping to protect individuals when neutralizing antibody levels induced by IIV are suboptimal. There was no evidence that lymphatic drainage patterns affected immunological outcomes, as results were consistent when OVX836 and IIVs were administered in the same arm as previously reported [19] or in different arms in the present study. In summary, concomitant OVX836 administration with licensed IIVs did not appear to adversely impact safety or immunogenicity of licensed influenza vaccines, while eliciting significant CMI and anti-NP IgG antibodies. Such responses could potentially enhance vaccine effectiveness, particularly in years of mismatch between IIVs and viral circulation. However, the present study was not designed to assess clinical efficacy, and the potential impact of these additional immune responses on protection against influenza warrants further investigation. Collectively, these findings support further clinical development of OVX836 in combination with licensed IIVs.

## Figures and Tables

**Figure 1:**
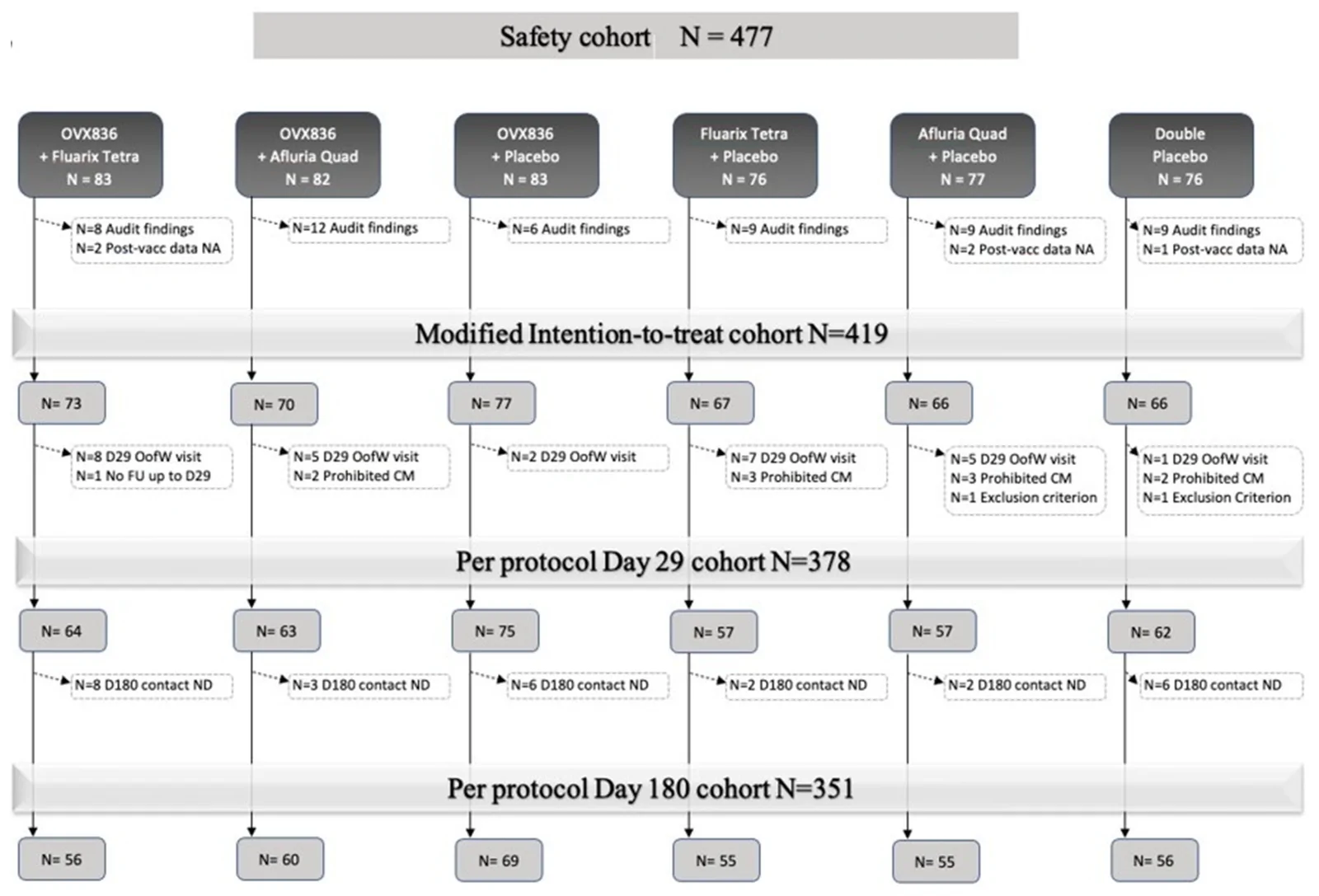
CONSORT diagram of the OVX836-006 study. CM = concomitant medication; FU = follow-up; NA = not available; ND = not done; OofW = outside of window.

**Figure 2:**
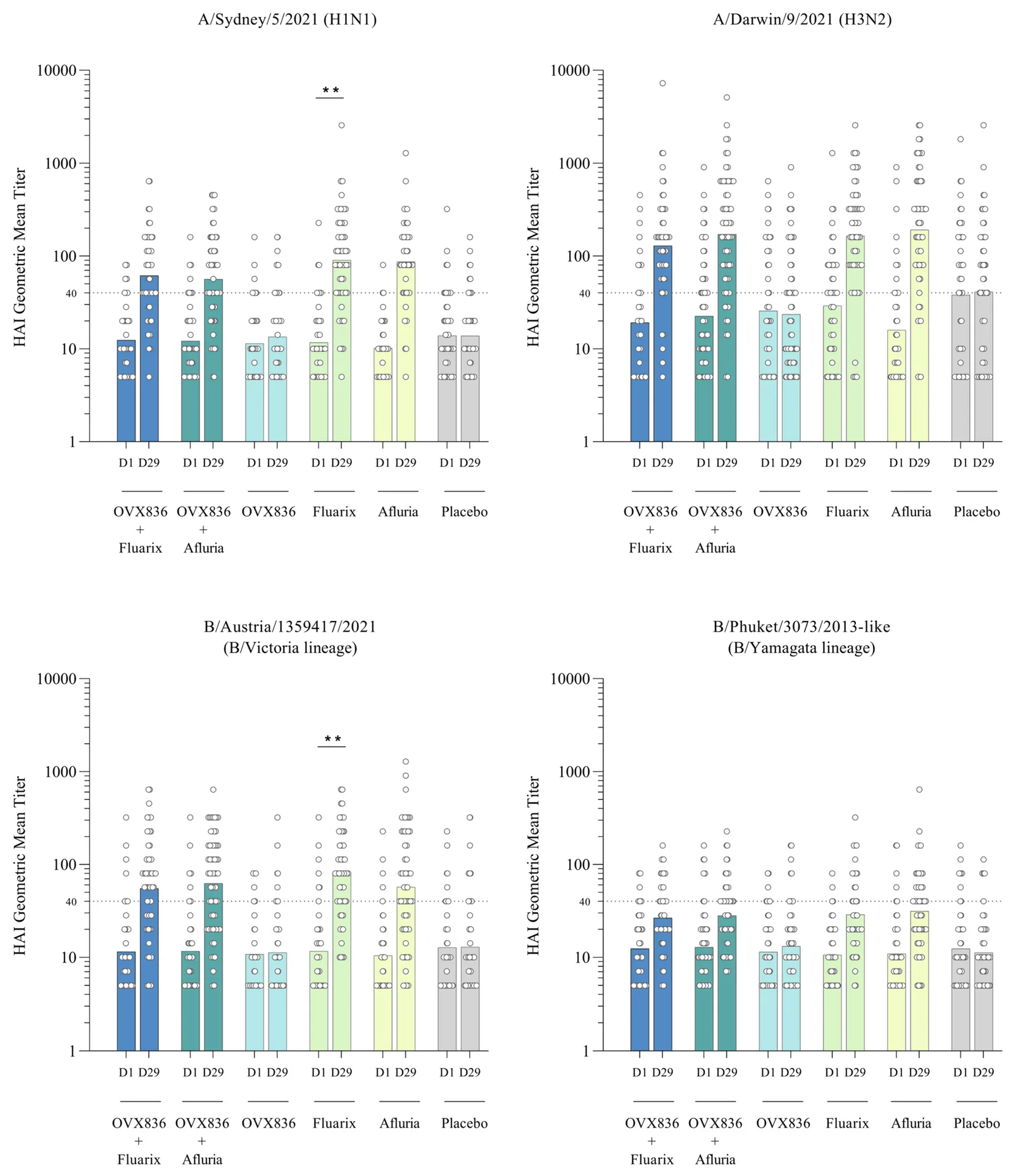
Hemagglutination inhibition geometric mean titers (GMTs) at baseline (Day 1) and Day 29 for the four influenza strains (per-protocol cohort). Results are shown as individual data and GMTs (horizontal bar). Intragroup comparisons = paired Student’s *t*-tests (** *p* < 0.01; paired Student’s *t* tests on log10 values, the dash line at the titer 40 corresponds to the seroprotection level).

**Figure 3:**
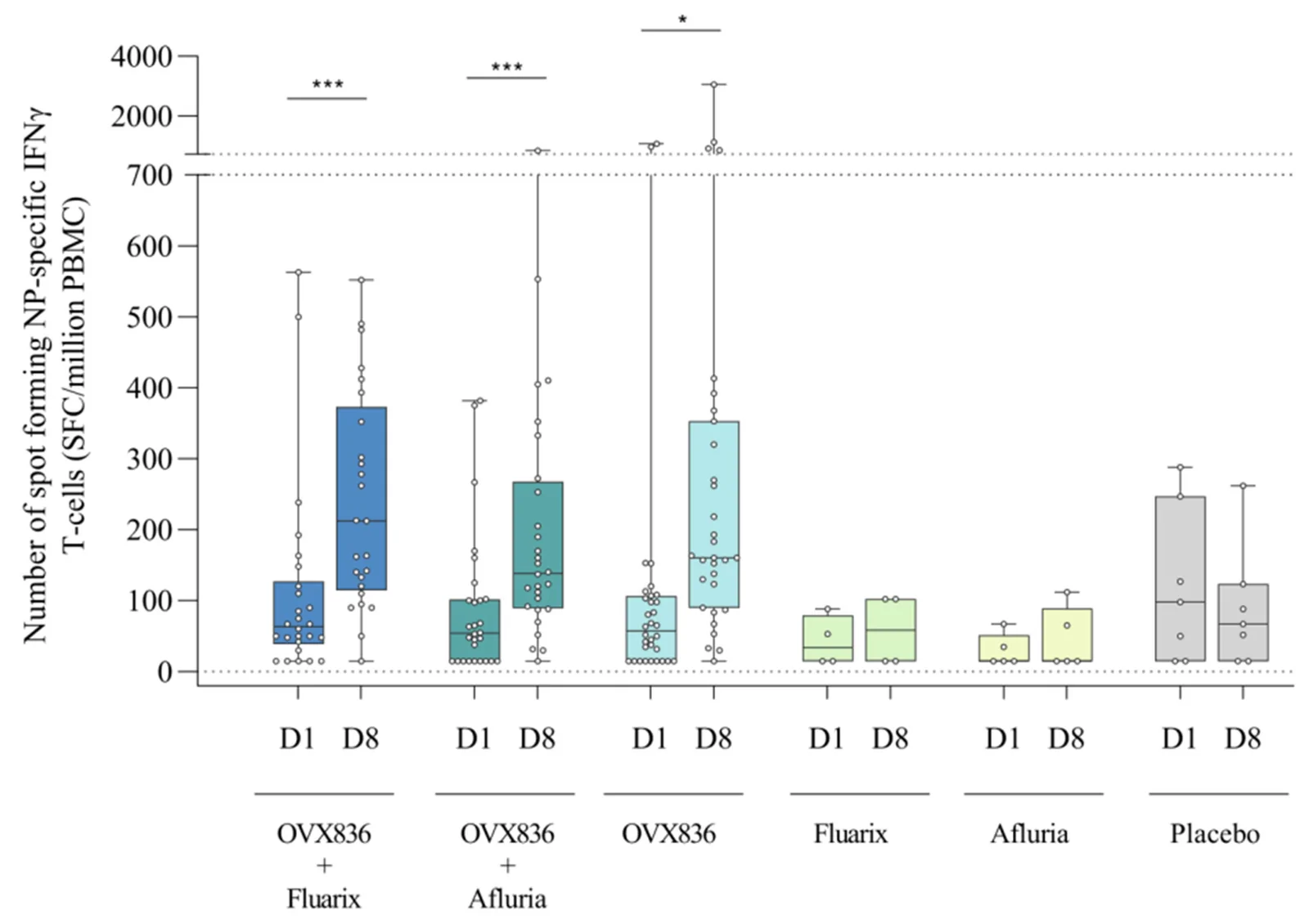
Box plot of the number of NP-specific IFNγ spot-forming cells (SFC/million PBMC) at Day 1 (pre-vaccination) and Day 8 (CMI subset of the per-protocol cohort). Results are shown as individual data, median (horizontal bar), interquartile interval (box), and minimum and maximum values. Intragroup differences * *p* < 0.05; *** *p* < 0.001 (paired Student’s *t* tests).

**Figure 4:**
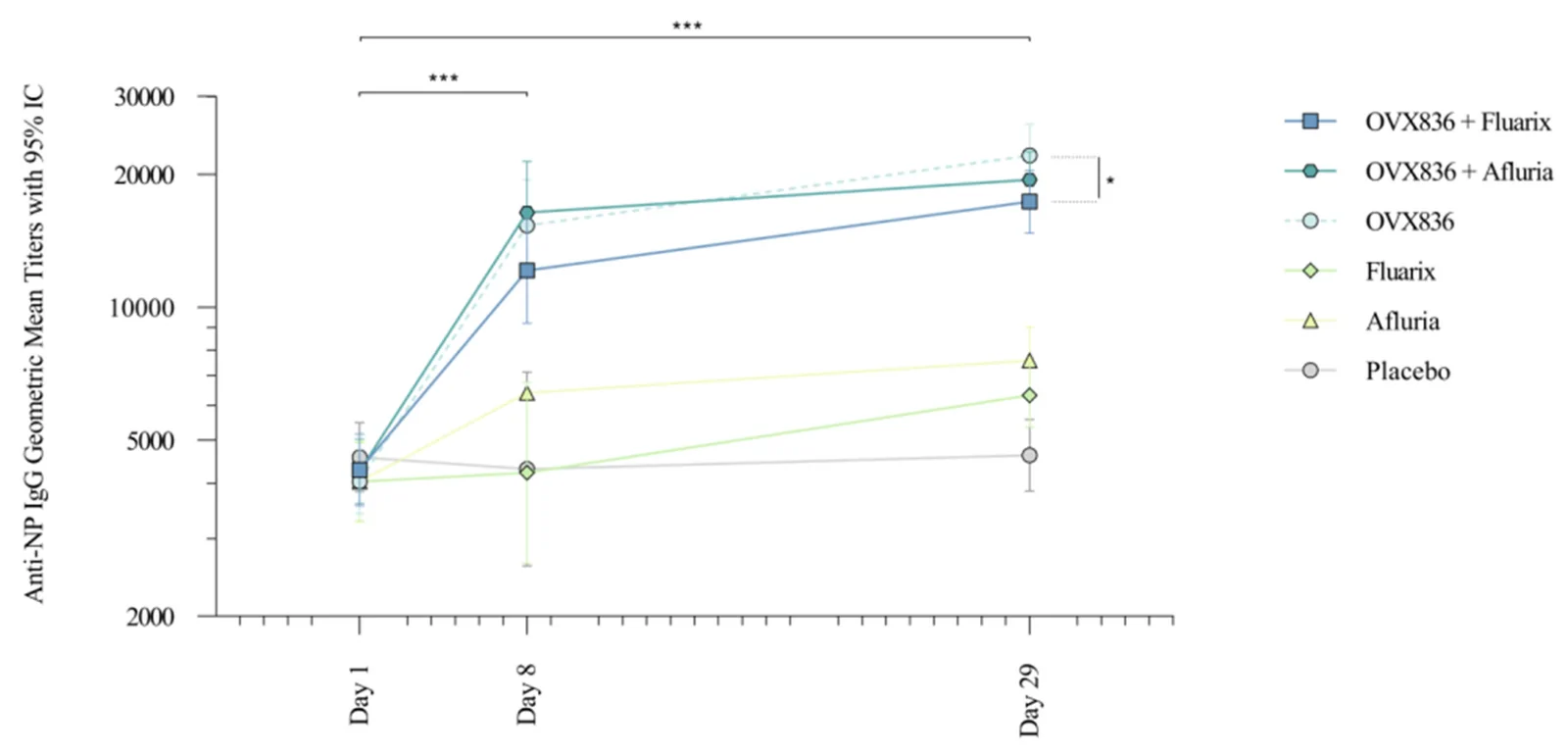
Anti-NP IgG titers at pre-injection baseline (Day 1), Day 8 and Day 29 (Days 1 and 29 = full per-protocol cohort; Day 8 = CMI subset of the per-protocol cohort). Results are given as geometric mean titers and 95% confidence intervals. Intragroup comparisons = paired Student’s *t* tests (*** *p* < 0.001). Intergroup comparisons = ANOVA followed by Bonferroni’s tests: * *p* = 0.039 for the difference between OVX836 + placebo and OVX836 + Fluarix.

**Figure 5:**
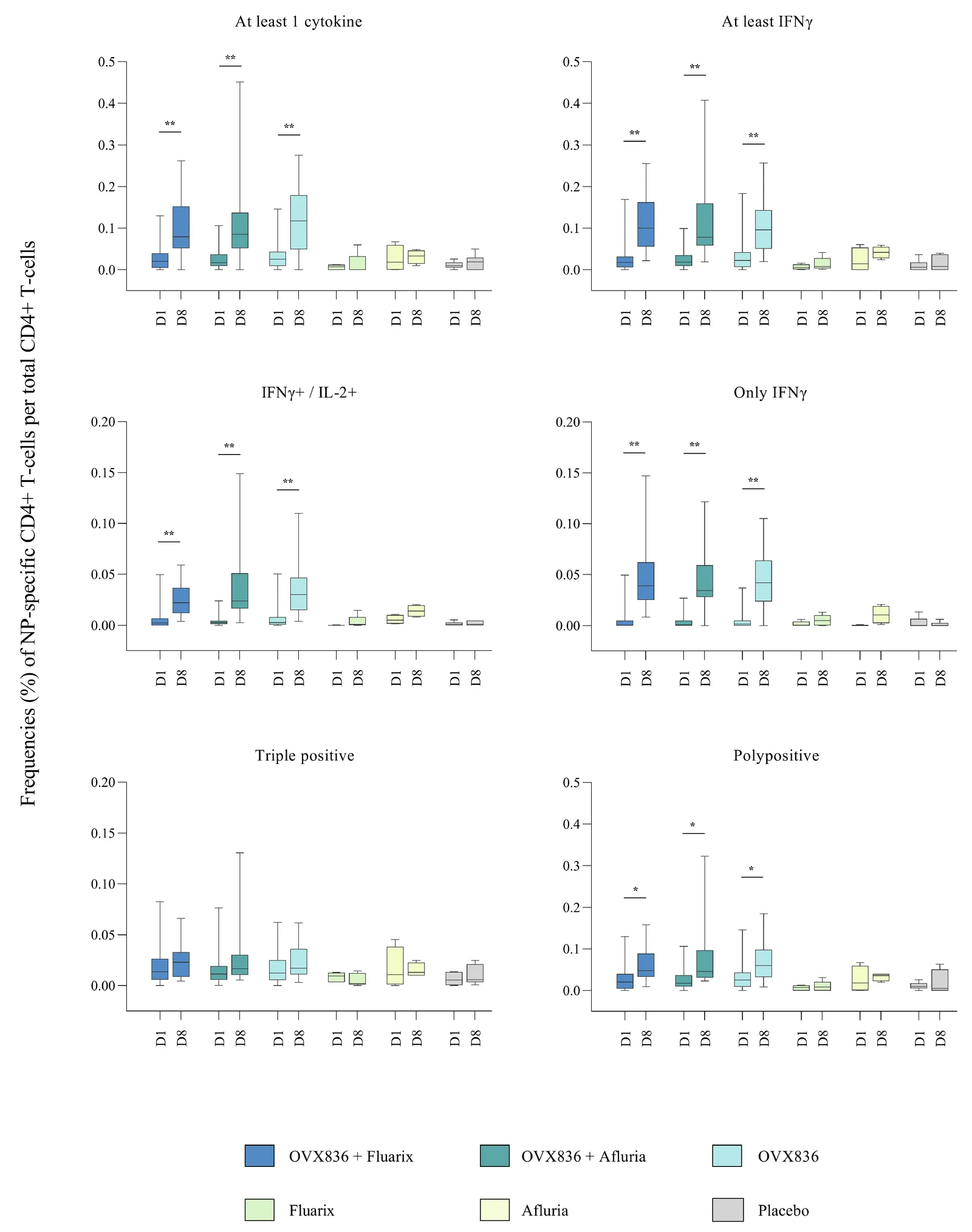
Box plot of the frequencies (%) of NP-specific CD4+ T cells per total CD4+ T cells positive for IFNγ, IL-2 and/or TNF at baseline (Day 1) and Day 8 in the six treatment groups (CMI subset of the per-protocol cohort). Results are given as median (horizontal bar), interquartile interval (box), minimum, and maximum values. Intragroup comparisons = Friedman’s tests (* *p* < 0.05; ** *p* < 0.01).

**Table 1:** Demographics and baseline characteristics of the study population, overall and by treatment group (safety set).

	OVX836 + Fluarix	OVX836 + Afluria	OVX836 + Placebo	Fluarix + Placebo	Afluria + Placebo	Double Placebo	Overall
	N = 83	N = 82	N = 83	N = 76	N = 77	N = 76	N = 477
Age (year)	39.5 ± 12.7	39.5 ± 13.3	38.6 ± 13.3	38.7 ± 12.8	37.0 ± 12.9	39.4 ± 12.4	38.8 ± 12.9
Weight (kg)	83.3 ± 17.3	82.9 ± 16.1	78.0 ± 16.1	82.9 ± 16.7	78.2 ± 14.8	81.0 ± 18.3	81.1 ± 16.6
Height (cm)	171 ± 10	172 ± 11	171 ± 9	172 ± 8	171 ± 10	172 ± 9	172 ± 10
BMI (kg/m^2^)	28.4 ± 4.8	28.1 ± 4.8	26.5 ± 4.3	27.9 ± 5.1	26.7 ± 4.4	27.1 ± 4.6	27.5 ± 4.7
Sex (female)	40 (48.2)	43 (52.4)	45 (54.2)	38 (50.0)	36 (46.8)	38 (50.0)	240 (50.3)
Race (White)	59 (71.1)	67 (81.7)	61 (73.5)	65 (85.5)	59 (76.6)	61 (80.3)	372 (78.0)
Smoking status							
Nonsmoker	63 (75.9)	59 (72.0)	58 (69.9)	52 (68.4)	58 (75.3)	52 (68.4)	342 (71.7)
Current	3 (3.6)	7 (8.5)	8 (9.6)	9 (11.8)	6 (7.8)	8 (10.5)	41 (8.6)
Former	17 (20.5)	16 (19.5)	17 (20.5)	15 (19.7)	13 (16.9)	16 (21.1)	94 (19.7)

Results are given as mean ± SD for continuous variables, and number (percentage) of subjects for discrete variables.

**Table 2:** Numbers (percentages) of participants with solicited local and systemic symptoms for 7 days post-administration and unsolicited adverse events 28 days post-administration (safety set).

	Intensity/Relatedness	OVX836 + Fluarix	OVX836 + Afluria	OVX836 + Placebo	Fluarix + Placebo	Afluria + Placebo	Double Placebo
		N = 83	N = 82	N = 83	N = 76	N = 77	N = 76
	All	70 (84.3)	69 (84.1)	72 (86.7)	65 (85.5)	62 (80.5)	49 (64.5)
	Grade ≥ 3	0 (0.0)	1 (1.2)	2 (2.4)	1 (1.3)	2 (2.6)	0 (0.0)
Solicited local symptoms							
	All	66 (79.5)	63 (76.8)	70 (84.3)	49 (64.5)	52 (67.5)	23 (30.3)
	Grade ≥ 3	0 (0.0)	0 (0.0)	0 (0.0)	1 (1.3)	0 (0.0)	0 (0.0)
Pain	All	64 (77.1)	63 (76.8)	70 (84.3.0)	49 (64.5)	52 (67.5)	22 (28.9)
	Grade ≥ 3	0 (0.0)	0 (0.0)	0 (0.0)	1 (1.3)	0 (0.0)	0 (0.0)
Redness	All	7 (8.4)	4 (4.9)	1 (1.2)	2 (2.6)	1 (1.3)	2 (2.6)
	Grade ≥ 3	0 (0.0)	0 (0.0)	0 (0.0)	0 (0.0)	0 (0.0)	0 (0.0)
Swelling	All	7 (8.4)	7 (8.5)	4 (4.8)	2 (2.6)	1 (1.3)	3 (3.9)
	Grade ≥ 3	0 (0.0)	0 (0.0)	0 (0.0)	0 (0.0)	0 (0.0)	0 (0.0)
Solicited systemic symptoms							
	All	50 (60.2)	45 (54.9)	51 (61.4)	58 (76.3)	50 (64.9)	45 (59.2)
	Grade ≥ 3	0 (0.0)	1 (1.2)	2 (2.4)	1 (1.3)	2 (2.6)	0 (0.0)
Arthralgia	All	15 (18.1)	11 (13.4)	13 (15.7)	13 (17.1)	7 (9.1)	11 (14.5)
	Grade ≥ 3	0 (0.0)	0 (0.0)	0 (0.0)	0 (0.0)	0 (0.0)	0 (0.0)
Fatigue	All	39 (47.0)	34 (41.5)	35 (42.2)	39 (51.3)	35 (45.5)	33 (43.4)
	Grade ≥ 3	0 (0.0)	1 (1.2)	0 (0.0)	0 (0.0)	0 (0.0)	0 (0.0)
Fever	All	1 (1.2)	2 (2.4)	0 (0.0)	1 (1.3)	1 (1.3)	0 (0.0)
	Grade ≥ 3	0 (0.0)	0 (0.0)	0 (0.0)	0 (0.0)	1 (1.3)	0 (0.0)
Headache	All	27 (32.5)	20 (24.4)	28 (33.7)	41 (53.9)	25 (32.5)	22 (28.9)
	Grade ≥ 3	0 (0.0)	0 (0.0)	1 (1.2)	1 (1.3)	0 (0.0)	0 (0.0)
Malaise	All	21 (25.3)	11 (13.4)	16 (19.3)	19 (25.0)	9 (11.7)	12 (15.8)
	Grade ≥ 3	0 (0.0)	0 (0.0)	1 (1.2)	0 (0.0)	0 (0.0)	0 (0.0)
Myalgia	All	34 (41.0)	26 (31.7)	30 (36.1)	31 (40.8)	24 (31.2)	10 (13.2)
	Grade ≥ 3	0 (0.0)	0 (0.0)	0 (0.0)	0 (0.0)	1 (1.3)	0 (0.0)
Unsolicited adverse events							
	All	17 (20.5)	23 (28.0)	22 (26.5)	22 (28.9)	24 (31.2)	22 (28.9)
	Grade ≥ 3	0 (0.0)	2 (2.4)	1 (1.2)	1 (1.3)	2 (2.6)	0 (0.0)
Related to IP	All	7 (8.4)	6 (7.3)	3 (3.6)	6 (7.9)	5 (6.5)	8 (10.5)
	Grade ≥ 3	0 (0.0)	1 (1.2) *	0 (0.0)	0 (0.0)	0 (0.0)	0 (0.0)
SAE	All	1 (1.2)	2 (2.4)	5 (6.0)	3 (3.9)	3 (3.9)	2 (2.6)
	Related to IP	0 (0.0)	0 (0.0)	0 (0.0)	0 (0.0)	0 (0.0)	0 (0.0)

* Paraesthesia; IP = investigational product.

**Table 3:** Summary of (i) the hemagglutination inhibition assay antibody response when Fluarix and Afluria were concomitantly administered with OVX836 or placebo at Day 29 and (ii) achievement of the Committee for Medicinal Products for Human Use (CHMP) and Food and Drug Administration (FDA) guideline criteria (per-protocol cohort).

Parameter	Strain	OVX836 + Fluarix (N = 64) n (%) [95% CI]	Fluarix + Placebo (N = 57) n (%) [95% CI]	OVX836 + Afluria (N = 63) n (%) [95% CI]	Afluria + Placebo (N = 57) n (%) [95% CI]
Seroprotection rate: CHMP criterion = 70% FDA criterion = lower limit of the 95% CI ≥ 70%	A/Sydney/5/2021 (H1N1)	49 (76.6) * [64.3–86.3]	48 (84.2) * [72.1–92.5] **	50 (79.4) * [67.3–88.5]	50 (87.7) * [76.3–94.9] **
	A/Darwin/9/2021 (H3N2)	59 (92.2) * [82.7–97.4] **	53 (93.0) * [83.0–98.1] **	54 (85.7) * [74.6–93.3] **	49 (86.0) * [74.2–93.7] **
	B/Austria/1359417/2021 (B/Victoria lineage)	43 (67.2) [54.3–78.4]	46 (80.7) * [68.1–90.0]	43 (68.3) [55.3–79.4]	38 (66.7) [52.9–78.6]
	B/Phuket/3073/2013-like (B/Yamagata lineage)	26 (40.6) [28.5–53.6]	22 (38.6) [26.0–52.4]	29 (46.0) [33.4–59.1]	38 (49.1) [35.6–62.7]
Seroconversion rate CHMP criterion = 40% FDA criterion = lower limit of the 95% CI ≥ 40%	A/Sydney/5/2021 (H1N1)	37 (57.8) * [44.8–70.1] **	42 (73.7) * [60.3–84.5] **	44 (69.8) * [57.0–80.8] **	45 (78.9) * [66.1–88.6] **
	A/Darwin/9/2021 (H3N2)	43 (67.2) * [54.3–78.4] **	42 (73.7) * [60.3–84.5] **	37 (58.7) * [45.6–71.0] **	36 (63.2) * [49.3–75.6] **
	B/Austria/1359417/2021 (B/Victoria lineage)	32 (50.0) * [37.2–62.8]	36 (63.2) * [49.3–75.6] **	35 (55.6) * [42.5–68.1] **	32 (56.1) * [42.4–69.3] **
	B/Phuket/3073/2013-like (B/Yamagata lineage)	10 (15.6) [7.8–26.9]	13 (22.8) [12.7–35.8]	16 (25.4) [15.3–37.9]	16 (28.1) [17.0–41.5]
		GMR (95% CI)	GMR (95% CI)	GMR (95% CI)	GMR (95% CI)
Day 29/Day 1 GMR CHMP criterion: GMR > 2.5 No FDA criterion	A/Sydney/5/2021 (H1N1)	4.97 * (3.72–6.64)	8.86 * (6.52–12.05)	5.94 * (4.58–7.71)	7.64 * (5.60–10.43)
	A/Darwin/9/2021 (H3N2)	7.37 * (5.23–10.38)	10.32 * (6.93–15.37)	5.87 * (4.34–7.95)	8.24 * (5.37–12.66)
	B/Austria/1359417/2021 (B/Victoria lineage)	4.79 * (3.53–6.49)	7.47 * (5.41–10.34)	5.35 * (3.93–7.26)	5.33 * (3.55–7.99)
	B/Phuket/3073/2013-like (B/Yamagata lineage)	2.13 (1.70–2.68)	2.63 * (2.06–3.35)	2.64 * (2.14–3.27)	2.75 * (2.02–3.73)

N = total number of subjects; n (%) = number and percentage of subjects; GMR = Geometric Mean Ratio; 95% CI = 95% confidence interval. * CHMP criterion achieved and ** FDA + CHMP criterion achieved (seroprotection rate and seroconversion rate only).

## Data Availability

The data presented in this study are available in this article and Supplementary Material. The results have also been published on the ClinicalTrials.gov website.

## Supplementary Materials

The following supporting information can be downloaded at: https://www.mdpi.com/article/10.3390/vaccines14100860/s1, Supplementary File S1: Eligibility criteria; Supplementary File S2: Immunoassays; Supplementary File S3: Statistical methods.

## Abbreviations

The following abbreviations are used in this manuscript:

AE	Adverse Event
CI	Confidence Interval
CMI	Cell-Mediated Immunity
GCP	Good Clinical Practice
GMR	Geometric Mean Ratio
GMT	Geometric Mean Titer
HA	Hemagglutinin
HAI	Hemagglutination Inhibition Assay
IFNγ	Interferon Gamma
IgG	Immunoglobulin G
IIV	Inactivated Influenza Vaccine
IL2	Interleukin 2
ILI	Influenza-Like Illness
IM	Intramuscular
LL	Lower Limit
mITT	Modified Intention-To-Treat
NA	Neuraminidase
NP	Nucleoprotein
PBMC	Peripheral Blood Mononuclear Cell
PP	Per Protocol
RT-PCR	Reverse Transcriptase-Polymerase Chain Reaction
SAE	Serious Adverse Event
SD	Standard Deviation
SFC	Spot-Forming Cell
TNFα	Tumor Necrosis Factor Alpha
UL	Upper Limit

## References

[1] World Health Organization (2025). Influenza (Seasonal) [Internet].

[2] Osterholm M.T., Kelley N.S., Sommer A., Belongia E.A. (2012). Efficacy and Effectiveness of Influenza Vaccines: A Systematic Review and Meta-Analysis. Lancet Infect. Dis..

[3] Nature Medicine (2018). A Checkup for the Flu Vaccine. Nat. Med..

[4] Trombetta C.M., Kistner O., Montomoli E., Viviani S., Marchi S. (2022). Influenza Viruses and Vaccines: The Role of Vaccine Effectiveness Studies for Evaluation of the Benefits of Influenza Vaccines. Vaccines.

[5] Bartsch S.M., O’Shea K.J., Ferguson M.C., Bottazzi M.E., Wedlock P.T., Strych U., McKinnell J.A., Siegmund S.S., Cox S.N., Hotez P.J. (2020). Vaccine Efficacy Needed for a COVID-19 Coronavirus Vaccine to Prevent or Stop an Epidemic as the Sole Intervention. Am. J. Prev. Med..

[6] Jang Y.H., Seong B.L. (2020). Call for a Paradigm Shift in the Design of Universal Influenza Vaccines by Harnessing Multiple Correlates of Protection. Expert Opin. Drug Discov..

[7] Mosmann T.R., McMichael A.J., LeVert A., McCauley J.W., Almond J.W. (2024). Opportunities and Challenges for T Cell-Based Influenza Vaccines. Nat. Rev. Immunol..

[8] Neumann G., Cowling B.J., Chen H., Stertz S., Manicassamy B., Barr I.G., Uyeki T.M., Kawaoka Y. (2026). Influenza. Nat. Rev. Dis. Primers.

[9] Epstein S.L., Kong W., Misplon J.A., Lo C.-Y., Tumpey T.M., Xu L., Nabel G.J. (2005). Protection against Multiple Influenza A Subtypes by Vaccination with Highly Conserved Nucleoprotein. Vaccine.

[10] Krammer F., Palese P. (2015). Advances in the Development of Influenza Virus Vaccines. Nat. Rev. Drug Discov..

[11] Whitmire J.K. (2011). Induction and Function of Virus-Specific CD4+ T Cell Responses. Virology.

[12] McKinstry K.K., Strutt T.M., Swain S.L. (2011). Hallmarks of CD4 T Cell Immunity against Influenza: Review: CD4 T Cell Immunity against Influenza. J. Intern. Med..

[13] Sridhar S., Begom S., Bermingham A., Hoschler K., Adamson W., Carman W., Bean T., Barclay W., Deeks J.J., Lalvani A. (2013). Cellular Immune Correlates of Protection against Symptomatic Pandemic Influenza. Nat. Med..

[14] Wilkinson T.M., Li C.K.F., Chui C.S.C., Huang A.K.Y., Perkins M., Liebner J.C., Lambkin-Williams R., Gilbert A., Oxford J., Nicholas B. (2012). Preexisting Influenza-Specific CD4+ T Cells Correlate with Disease Protection against Influenza Challenge in Humans. Nat. Med..

[15] Hofmeyer T., Schmelz S., Degiacomi M.T., Dal Peraro M., Daneschdar M., Scrima A., Van Den Heuvel J., Heinz D.W., Kolmar H. (2013). Arranged Sevenfold: Structural Insights into the C-Terminal Oligomerization Domain of Human C4b-Binding Protein. J. Mol. Biol..

[16] Del Campo J., Pizzorno A., Djebali S., Bouley J., Haller M., Pérez-Vargas J., Lina B., Boivin G., Hamelin M.-E., Nicolas F. (2019). OVX836 a Recombinant Nucleoprotein Vaccine Inducing Cellular Responses and Protective Efficacy against Multiple Influenza A Subtypes. npj Vaccines.

[17] Leroux-Roels I., Willems P., Waerlop G., Janssens Y., Tourneur J., De Boever F., Bruhwyler J., Alhatemi A., Jacobs B., Nicolas F. (2023). Immunogenicity, Safety, and Preliminary Efficacy Evaluation of OVX836, a Nucleoprotein-Based Universal Influenza A Vaccine Candidate: A Randomised, Double-Blind, Placebo-Controlled, Phase 2a Trial. Lancet Infect. Dis..

[18] Jacobs B., Leroux-Roels I., Bruhwyler J., Groth N., Waerlop G., Janssens Y., Tourneur J., De Boever F., Alhatemi A., Moris P. (2024). Evaluation of Safety, Immunogenicity and Cross-Reactive Immunity of OVX836, a Nucleoprotein-Based Universal Influenza Vaccine, in Older Adults. Vaccines.

[19] Groth N., Bruhwyler J., Tourneur J., Piat E., Moris P., Le Vert A., Nicolas F. (2025). Safety and Immunogenicity of OVX836, a Nucleoprotein-Based Universal Influenza Vaccine, Co-Administered with Fluarix® Tetra, a Seasonal Hemagglutinin-Based Vaccine. Vaccines.

[20] FDA Fluarix Product Information. https://www.fda.gov/vaccines-blood-biologics/vaccines/fluarix.

[21] Miller M.S., Montomoli E., Leshem E., Schotsaert M., Weinke T., Vicic N., Rudin D. (2024). Seasonal Influenza Vaccines: Variability of Immune Responses to B Lineage Viruses. Hum. Vaccines Immunother..

[22] Reis L.R., Silva-Moraes V., Teixeira-Carvalho A., Ross T.M. (2024). B-Cell Dynamics Underlying Poor Response upon Split-Inactivated Influenza Virus Vaccination. Front. Immunol..

[23] Xu Q., Wei H., Wen S., Chen J., Lei Y., Cheng Y., Huang W., Wang D., Shu Y. (2023). Factors Affecting the Immunogenicity of Influenza Vaccines in Human. BMC Infect. Dis..

[24] World Health Organization FluNet Summary [Internet]. https://www.who.int/tools/flunet/flunet-summary.

[25] Ko Y.-A., Yu Y.-H., Wu Y.-F., Tseng Y.-C., Chen C.-L., Goh K.-S., Liao H.-Y., Chen T.-H., Cheng T.-J.R., Yang A.-S. (2021). A Non-Neutralizing Antibody Broadly Protects against Influenza Virus Infection by Engaging Effector Cells. PLoS Pathog..

[26] Oftung F., Næss L.M., Laake I., Stoloff G., Pleguezuelos O. (2022). FLU-v, a Broad-Spectrum Influenza Vaccine, Induces Cross-Reactive Cellular Immune Responses in Humans Measured by Dual IFN-γ and Granzyme B ELISpot Assay. Vaccines.

[27] Hao S., Tomic I., Lindsey B.B., Jagne Y.J., Hoschler K., Meijer A., Carreño Quiroz J.M., Meade P., Sano K., Peno C. (2025). Integrative Mapping of Preexisting Influenza Immune Landscapes Predicts Vaccine Response. J. Clin. Investig..

