# Supplementary material for: Double-Blind, Randomized, Phase 2a Study to Evaluate the Safety and Immunogenicity of OVX836, a Nucleoprotein-Based Universal Influenza Vaccine, Co-Administered with Fluarix Tetra® or Afluria Quad®
Source: Vaccines (Basel). 2026 Sep 28;14(10):860. doi: 10.3390/vaccines14100860 (PMC13709431; doi:10.3390/vaccines14100860)
Supplement: Supplementary file 1 [file vaccines-14-00860-s001.zip › OVX836-006_Paper for Vaccines_Supplementary S1.pdf]

Supplementary S1: Eligibility criteria

Inclusion criteria

The following criteria were checked at the time of screening (Day 1). The subject could only be included in the study if all inclusion criteria were fulfilled:

1. Provided written informed consent.
2. Healthy male or female subjects, as determined by medical history and medical examination.
3. Aged 18 to 60 years.
4. Subjects who had received at least two doses of a licensed SARS-CoV-2 (COVID-19) vaccine.
5. Reliable and willing to make themselves available for the duration of the study, and willing and able to follow study procedures.
6. Able to read, understand, and complete an eDiary and ePRO; availability of a person who can complete the eDiary/ePRO in case of illness.

Exclusion criteria

The following criteria were checked at the time of screening (Day 1). If any exclusion criterion applied, individuals could not be included in the study. Individuals with temporary exclusion criteria could be enrolled at a later point in time when the exclusion criterion had resolved:

1. Individuals with a body mass index (BMI) $\leq$19 kg/m^2 or $\geq$40 kg/m^2 on the day of vaccination.
2. Previous influenza vaccination within 6 months before the day of vaccination or planned influenza vaccination during the whole study period.
3. Any known or suspected immunodeficient conditions.
4. Past or current history of significant autoimmune diseases, as judged by the Investigator.
5. Known or suspected infection with human immunodeficiency virus (HIV), hepatitis C virus (HCV), or hepatitis B virus (HBV).
6. Current history of significant uncontrolled medical illness such as diabetes, hypertension, heart, renal or hepatic diseases, as judged by the Investigator.
7. Planned, recent (<6 months since completion) or ongoing gender reassignment during the study.
8. Females: pregnant, breastfeeding or of childbearing potential without appropriate contraceptive methods in place for 2 months before enrollment, or with a positive pregnancy test on the day of vaccination. Appropriate contraceptive methods are to be maintained until the end of the trial. Appropriate contraceptive methods are defined by the Clinical Trial Facilitation Group [CTFG] as follows: "Contraceptive methods that can achieve a failure rate of less than 1% per year

when used consistently and correctly are considered as highly effective birth control methods. Such methods included: combined (estrogen- and progestogen-containing) hormonal contraception associated with inhibition of ovulation (oral, intravaginal, transdermal), progestogen-only hormonal contraception associated with inhibition of ovulation (oral, injectable, implantable intrauterine device, intrauterine hormone-releasing system), bilateral tubal occlusion, vasectomized partner and/or sexual abstinence (refraining from heterosexual intercourse)."

9. Having received another vaccination within 3 months prior to the day of study vaccination for live attenuated vaccines, or within 1 month prior to the day of study vaccination for inactivated vaccines, except for the COVID-19 vaccine.
10. Planning to receive other vaccines during the first 28 days following the study vaccine administration.
11. Having received a COVID-19 vaccination within 2 weeks prior to the day of study vaccination.
12. Planning to receive a COVID-19 vaccine during the first week (within 7 days) following the study vaccine administration. An interval of preferably 14 days is recommended.
13. Administration of any investigational (including OVX836) or non-registered drug or vaccine within 3 months prior to the administration of study vaccines, or planned administration of any such product during the whole study period.
14. History of receiving blood, blood components, or immunoglobulins within 3 months prior to the day of vaccination, or planned to receive such a product during the whole study period.
15. Presence of an acute febrile illness on the day of planned vaccination (oral temperature >38.0°C; temporary exclusion criterion).
16. Long COVID, either ongoing or recently recovered.
17. Presence of a condition in the ear-nose-throat area, such as nasal septum deviation, atrophic rhinitis, etc., that could render nasal and nasopharyngeal swabs more difficult to perform, or increase the risk of bleeding; to be confirmed by a medical history question and inspection of the nasal passage.
18. Presence of tattoos at the level of one of the deltoid muscles.
19. Past or current history of any progressive or severe uncontrolled neurological disorder, seizure disorder, or Guillain-Barré syndrome.
20. Behavioral or cognitive impairment, or psychiatric disease that, in the opinion of the Investigator, may interfere with the individual's ability to participate in the study.
21. Past (stopped less than 6 months before enrollment), or current, smoking habit above 10 cigarettes per day.
22. Past (stopped less than 6 months before enrollment), or current, history of alcohol abuse or use of recreational drugs.
23. Treatment that can affect immune response such as systemic or high-dose inhaled corticosteroids (>800μg/day beclomethasone or equivalent; occasional inhaled corticosteroids for asthma therapy are allowed), radiation treatment, cytotoxic drugs, or current or recent (within 30 days before study entry) chronic or

prolonged (>10 days) use of systemic non-steroidal anti-inflammatory drugs (NSAIDs), acetylsalicylic acid, paracetamol, ibuprofen, interferon, immunomodulators, allergy shots, as judged by the Investigator. Occasional, non-continuous use of acetylsalicylic acid, paracetamol, ibuprofen or NSAIDs on an as-needed basis was allowed.

24. Prophylactic or therapeutic use of any anti(retro)virals by systemic route during the study. Topical application was allowed.
25. History of severe allergic reactions and/or anaphylaxis, or serious adverse reactions to vaccines, or allergy to kanamycin, eggs (especially ovalbumin and chicken proteins), neomycin, polymyxin, formaldehyde and octoxinol-9 (Triton-X-100).
26. Any contraindication to IM administration, as judged by the Investigator.
27. Individuals with a history of any illness that, in the opinion of the Investigator, might interfere with the results of the study, or pose additional risk due to participation in the study, either directly or through any treatments administered for that illness.
28. Sponsor employees or Investigator site personnel directly affiliated with this study, and their immediate families. Immediate family was defined as a spouse (or assimilated), parent, child, or sibling, whether biological or legally adopted.
