# Supplementary material for: Double-Blind, Randomized, Phase 2a Study to Evaluate the Safety and Immunogenicity of OVX836, a Nucleoprotein-Based Universal Influenza Vaccine, Co-Administered with Fluarix Tetra® or Afluria Quad®
Source: Vaccines (Basel). 2026 Sep 28;14(10):860. doi: 10.3390/vaccines14100860 (PMC13709431; doi:10.3390/vaccines14100860)
Supplement: Supplementary file 1 [file vaccines-14-00860-s001.zip › OVX836-006_Paper for Vaccines_Supplementary S2.pdf]

Supplementary S2: Immunoassays

1) **Peripheral blood mononuclear cell (PBMC) processing:** At the selected time points, ≈ 50 mL of blood was collected by venous puncture in heparin-coated blood collection tubes (Becton Dickinson Vacutainer tubes of 10 mL coated with lithium heparin, or equivalent). PBMCs were processed within 6 hours after blood collection. Briefly, after a 1:2 sample dilution in Dulbecco's phosphate-buffered saline (dPBS), PBMCs were isolated by density gradient centrifugation (Ficoll-Paque contained within SepMate™ tubes), washed three times in dPBS, suspended in freezing solution (CryoStor® CS10 cryopreservation medium, STEMCELL Technologies), frozen at a concentration of 6 to 20 million cells/vial, and stored in liquid nitrogen until use in the subsequent dedicated assays.

2) **ELISpot assay for peripheral blood mononuclear cells (PBMCs):** The ELISpot method was used to measure the number of PBMCs that secreted IFNγ upon NP re-stimulation (NP-specific spot-forming cells, SFCs). After PBMC thawing, resuspension in culture medium, and overnight resting, 2 x 10^5 PBMCs/well in triplicate wells were incubated for 24 hours with the NP peptide pool (>80% purity), at a final concentration of 4µg per peptide/mL. The NP peptide pool was composed of 122 x 15-mer peptides overlapping by 11 amino acids and spanning the amino acid sequence of the NP from the influenza A/WSN/33 (H1N1) strain. As a negative control, cells were incubated under the same conditions without the peptide pool (culture medium + 0.3% dimethyl sulfoxide). The average number of SFCs for the negative control was subtracted from the average number of SFCs for the test wells, and results were reported as SFCs per million PBMCs for each clinical sample. The ELISpot assay was qualified using quality control (QC) samples. The Limit of Detection (LOD) was 29 SFC/million PBMCs, and the estimated lower limit of quantification (LLOQ) was 82 SFC/million PBMCs. The estimated upper limit of quantification (ULOQ) was 3743 SFC/million PBMCs (based on an anti-CD3 positive control, as no sample exhibiting high NP signal was available for the assay qualification). The method presented acceptable within- and between-run precision for samples >LLOQ (coefficient of variation [CV] within-run was <30%, CV between-run <50% on at least 3 days, with 3 replicates of triplicate wells per day). One internal QC was tested on each plate in parallel with the evaluation of clinical samples to validate the plate according to predefined criteria (if criteria were not fulfilled, the plate was not valid and a retest was performed). Data below the LOD on clinical samples were reported as 15 SFC/million PBMCs (1/2 LOD) for the statistical analyses.

3) **Flow cytometry – Intracellular staining (ICS) for PBMCs:** An adaptation of the method described by Moris et al. (2011, J. Clin. Immunol. 31(3), 443-454) was used to measure NP-specific $CD4^+$ and $CD8^+$ T cells positive for interleukin 2 (IL2), tumor necrosis factor alpha (TNFα) and interferon gamma (IFNγ). After thawing, the cells were washed with culture medium and incubated *in vitro* with the relevant antigen (NP, C4BP or OVX313) versus corresponding medium in the presence of costimulatory antibodies to CD28 and CD49d for two hours. Then Brefeldin A, a cytokine secretion blocker, was added for a subsequent overnight stimulation, ensuring the inhibition of cytokine secretion and its accumulation in the expressing cells. The day after, the cells were

stained using fluorochrome-conjugated antibodies to phenotypic markers (CD3, CD4 and CD8) and cytokines (IFNγ, IL2 and TNFα) (antibodies and fluorochromes are shown in the table below). The samples were then analyzed using a BD Fortessa X-20 flow cytometer. The relevant antigen was the same peptide pool used in the ELISpot assay and consisted of 122 x 15-mer peptides overlapping by 11 amino acids and spanning the sequence of the NP from influenza A/WSN/33 (H1N1) strain. The concentration of stimulation was 4µg/mL of each peptide, and a negative control including 0.3% dimethyl sulfoxide (DMSO) was tested for each sample. The NP-specific $CD4^+$ and $CD8^+$ T cells were determined by flow cytometry as the $CD3^+CD4^+$ and $CD3^+CD8^+$ events expressing one cytokine or a combination of cytokines among IFNγ, IL2, and TNFα after *in vitro* stimulation with relevant antigen, subtracted by the corresponding signal of the same sample obtained after *in vitro* stimulation with medium + DMSO (background). The ICS results were reported as the frequencies (%) of NP-specific $CD4^+$ or $CD8^+$ T cells per $CD4^+$ T cells and CD8+ T-cell parent populations, respectively.

Fluorochrome-conjugated antibodies used in ICS assay

Reagent specificity	Clone	Conjugated dye	Catalog	Provider
CD3	UCHT1	AF700	557943	Becton Dickinson
CD4	SK3	PerCP	345770	Becton Dickinson
CD8	SK1	APC-H7	641400	Becton Dickinson
IFNγ	4S.B3	FITC	554551	Becton Dickinson
TNFα	Mab11	PE-Cy7	557647	Becton Dickinson
IL2	MQ1-17H12	APC	554567	Becton Dickinson

4) **Assay for anti-NP serum IgG antibody:** The method used to measure anti-NP serum IgG antibody was an indirect ELISA including the following steps: (i) coating of NP (Osivax R&D batch) on the wells of the plate, (ii) blocking of non-specific sites using bovine serum albumin, (iii) incubation with the clinical samples after two-fold serial dilutions (first dilution 1:800), (iv) addition of the secondary anti-human IgG antibody conjugated to horseradish peroxidase (ThermoFisher, A18805, dilution 1:40,000), (v) revelation of the signal using 3,3',5,5'-tetramethylbenzidine (TMB) substrate, and (vi) blocking the reaction with a stop solution before reading the plate at 450 nm. The anti-NP IgG titer for a serum sample was defined as the highest tested dilution giving an optical density >2 fold the signal of the negative control tested on each plate. The method was qualified using different positive (QC+) and negative (QC-) serum controls. The method was specific, selective, and demonstrated acceptable within-run precision (n=6, CV <20% on optical densities; QC+ titer: 6400 ± 1 dilution) and between-run precision (CV <30% on optical densities; QC+ titer: 6400 ± 1 dilution, n=3 tests on 3 different days with 2 operators). The stability of the QC samples after 5 freeze-thaw cycles and their stability after 5 months at -20°C were also checked. The QC samples were used in parallel with clinical samples to validate each plate according

to predefined criteria (if criteria were not fulfilled, the plate was considered not valid and a retest was performed).

5) **Serum hemagglutination-inhibition assay:** The serum hemagglutination-inhibition (HAI) assay is the most used reference method for the assessment of anti-HA antibody levels (Waldock et al., 2021). The method is based on the ability of the influenza virus to agglutinate red blood cells (RBCs) and the ability of anti-HA antibodies to inhibit this agglutination. The biological relevance of the assay is rooted in the fact that the agglutination is mediated by the receptor-binding site of the HA1 domain, which is also a common target for neutralizing antibodies (Sawant et al., 2023). In brief, serum samples are treated with a receptor-destroying enzyme to remove nonspecific agglutination that may occur. Serum sample dilutions are then mixed with a standardized amount of HA (4 hemagglutination assay units), and the hemagglutination inhibition titer is expressed as the reciprocal of the highest serum dilution able to prevent the hemagglutination of the RBCs. HAI seroconversion is assessed by comparing the inhibition titer post-vaccination to the pre-vaccination titer, and was actually defined as a negative pre-vaccination HAI titer and a post-vaccination HAI titer ≥1:40, or a fourfold increase in HAI titer between pre- and post-vaccination timepoints (according to the Note for Guidance on Harmonization of Requirements for Influenza Vaccines, CPMP/BWP/214/96, 12th March 1997). Relevant and pre-established positive and negative QC serum samples were included in the testing runs and used to certify the results. The assay was validated before initiating clinical sample testing. The parameters examined were: linearity, relative accuracy, precision/repeatability (intra-assay precision), intermediate precision, limit of quantitation and range, and specificity. The validation experiments were performed for the following viruses, corresponding to the strains included in the seasonal influenza vaccine used in the study:

- A/Sydney/5/2021 (H1N1) pdm09-like virus
- A/Darwin/9/2021 (H3N2)-like virus
- B/Austria/1359417/2021 (B/Victoria lineage)-like virus
- B/Phuket/3073/2013 (B/Yamagata lineage)-like virus

Using selected sheep or ferret sera as positive sera against the relevant strains and human serum depleted of IgA/IgM/IgG as a negative control, the HAI assay validation was found to be linear for (i) A/Sydney/5/2021 (H1N1) pdm09-like strain, up to the 1:512 dilution, for (ii) A/Darwin/9/2021 (H3N2)-like strain, up to the 1:1024 dilution, (iii) B/Austria/1359417/2021 (B/Victoria lineage)-like strain, up to the 1:256 dilution, and (iv) B/Phuket/3073/2013 (B/Yamagata lineage)-like strain, up to the 1:128 dilution. The relative accuracy (defined as the agreement between the expected and observed titers across the dilution series) was within 50% - 200% of the expected titer, repeatability (% of geometric coefficient of variation [GCV]<65.5%), and intermediate precision (% of GCV<129%) were demonstrated for the four strains. The limits of

quantification and assay ranges were established. The performance of the HAI assay is detailed in the corresponding validation report.

6) **Laboratory confirmation of influenza**: In the case of ILI, RT-PCR commercial kits were used, in accordance with the label, to detect and confirm any infections by an influenza A or B strain, by RSV, or by SARS-CoV-2 on nasopharyngeal and nasal swab samples. Influenza A/B and RSV were detected using the Panther Fusion Flu A/B/RSV assay, which is a multiplex real-time PCR (RT-PCR) in vitro diagnostic test for rapid and qualitative detection and differentiation of influenza A virus, influenza B virus, and respiratory syncytial virus (RSV). The Panther Fusion® SARS-CoV-2 Assay is a real-time RT-PCR in vitro diagnostic test that was used for the qualitative detection of RNA from SARS-CoV-2 isolated and purified from nasopharyngeal swab specimens.
