# Supplementary material for: Double-Blind, Randomized, Phase 2a Study to Evaluate the Safety and Immunogenicity of OVX836, a Nucleoprotein-Based Universal Influenza Vaccine, Co-Administered with Fluarix Tetra® or Afluria Quad®
Source: Vaccines (Basel). 2026 Sep 28;14(10):860. doi: 10.3390/vaccines14100860 (PMC13709431; doi:10.3390/vaccines14100860)
Supplement: Supplementary file 1 [file vaccines-14-00860-s001.zip › OVX836-006_Paper for Vaccines_Supplementary S3.pdf]

Supplementary S3: Statistical methods

Sample size

To gauge the immune responses against HA elicited by IIVs against the regulatory threshold criteria for seasonal influenza vaccine approval, relevant regulatory guidance documents recommend the evaluation of 50 subjects (aged 18 to 60 years) per group (Committee for Proprietary Medicinal Products, Note for Guidance on Harmonization of Requirements for Influenza Vaccines, 12 March 1997, CPMP/BWP/214/96).

The primary objective of the protocol was initially to demonstrate achievement of the criteria for acceptance of seasonal influenza vaccines in two separate age strata (18-44 years and 45-60 years). Therefore, a total sample size of 100 subjects per treatment group (50 subjects in each age stratum) was to be included and vaccinated, i.e., a total of 600 subjects overall.

As the actual sample size was equal to 478 subjects, the primary endpoints of the study were analyzed in strict adherence to the above-mentioned European guidance, i.e., in a total of 50 evaluable subjects per treatment group on Day 29. The same parameters were not reanalyzed in the two age strata as initially planned (292 subjects aged 18-44 years and 186 subjects aged 45-60 years).

General statistical principles
SAS (Version 9.4) was used to perform all the statistical analyses. Missing values were not replaced or extrapolated.

Continuous variables: Descriptive statistics included the number of observations (n), arithmetic or geometric mean (titers [GMTs] or ratios [GMRs]) along with corresponding 95% confidence interval (CI), standard deviation (SD) for arithmetic mean only, median, minimum and maximum.

Categorical variables: Descriptive statistics included counts, percentages per category, and 95% CIs. The denominator in all percentage calculations was the number of subjects in the relevant analysis population with non-missing data.

Analysis of demographics and baseline characteristics
Demographics and baseline study population characteristics were described using descriptive statistics (as mentioned above). The six groups were compared using one-way ANOVA for continuous variables, or chi-square, Fisher's exact tests, or Mann–Whitney tests (as appropriate) for discrete variables.

Analysis of safety
Safety parameters were assessed using descriptive statistics (as mentioned above). Intergroup comparisons were performed using chi-square tests, Fisher's exact tests, or Mann–Whitney tests, as appropriate.

Analysis of immunogenicity
Immunogenicity parameters were evaluated using descriptive statistics (as mentioned above). Seroconversion rates, proportions of subjects achieving HAI titers ≥1:40 and GMRs (primary endpoints) were accompanied by their 95% exact CIs. Taking into account the central limit theorem and the fact that approximately 50 to 80 subjects were evaluable per group (>30 in all

cases), parametric tests were used for all inferential tests. In the PBMC subset, the central limit theorem was not applicable due to a lower number of subjects ($n<30$). In this case, the normality of continuous variables was tested using the Kolmogorov–Smirnov test. If normality was achieved, intergroup comparisons were performed using one-way ANOVA, followed when significant by post hoc Bonferroni's tests (adjusted for multiplicity, α threshold depending on the number of treatment groups analyzed). If normality was not achieved, intergroup comparisons were performed using a Kruskal–Wallis test, followed when significant by post hoc Mann–Whitney tests (adjusted for multiplicity). Titers were analyzed the same way, after log10 transformation. Intergroup comparisons of discrete variables (fold-increase percentages) were performed using Fisher's exact tests. Intragroup comparisons were performed using ANOVA for repeated measures, followed, when significant, by post hoc paired t-tests adjusted for multiplicity (Bonferroni) for continuous and normal variables, or using Friedman's tests, followed, when significant, by post hoc paired Wilcoxon's tests for continuous and non-normal variables or for discrete variables.
